# Supplementary material for: CRISPR-mediated gene silencing reveals involvement of the archaeal S-layer in cell division and virus infection
Source: Nat Commun. 2019 Oct 22;10:4797. doi: 10.1038/s41467-019-12745-x (PMC6805947; doi:10.1038/s41467-019-12745-x)
Supplement: Supplementary file 1 — Supplementary Information [file 41467_2019_12745_MOESM1_ESM.pdf]

Supplementary Information

"CRISPR-mediated gene silencing reveals involvement of the archaeal S-layer in cell division and virus infection"

Zink and Pfeifer *et al.*

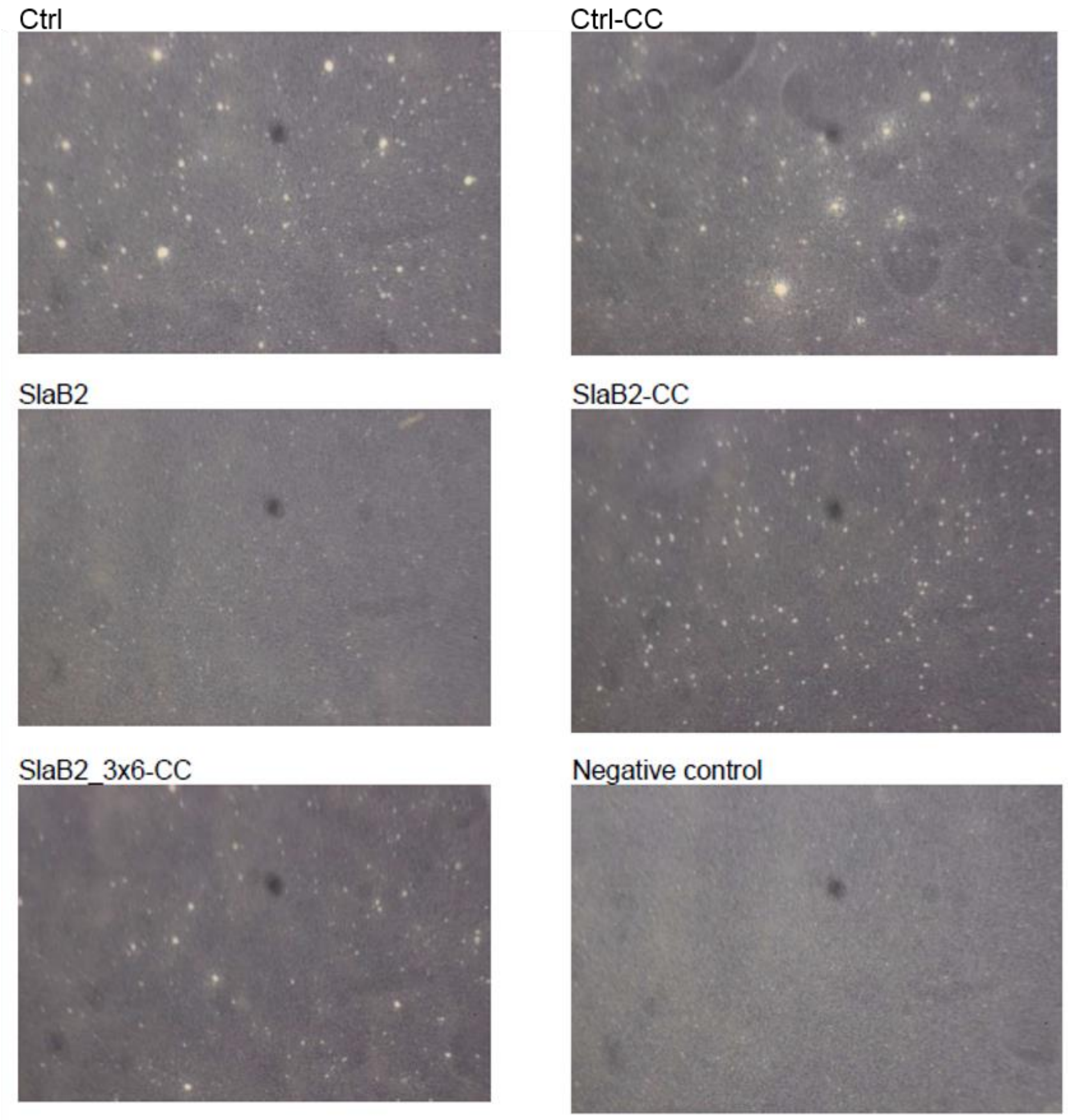

Supplementary Figure 1: Single colonies of pIZ-plasmid transformants after overlay plating. Colonies were formed after 10 days incubation. Cultures carrying the respective plasmid are indicated. CC= pIZ-SB complementation constructs.

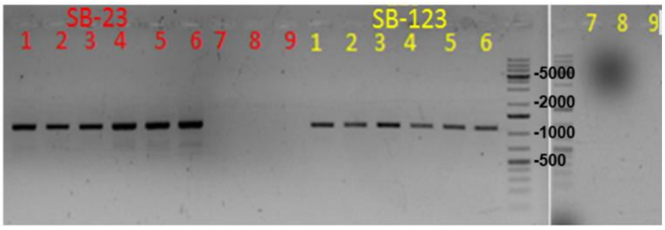

Supplementary Figure 2: Culture PCR, with primers ORF-904FW/RV binding the plasmid backbone, show loss of whole plasmid in plasmid SB23 and SB123 pIZ-plasmid primary cultures after 7 days incubation. Ladder: 1kb plus DNA Ladder, GeneRuler (Thermo Scientific).

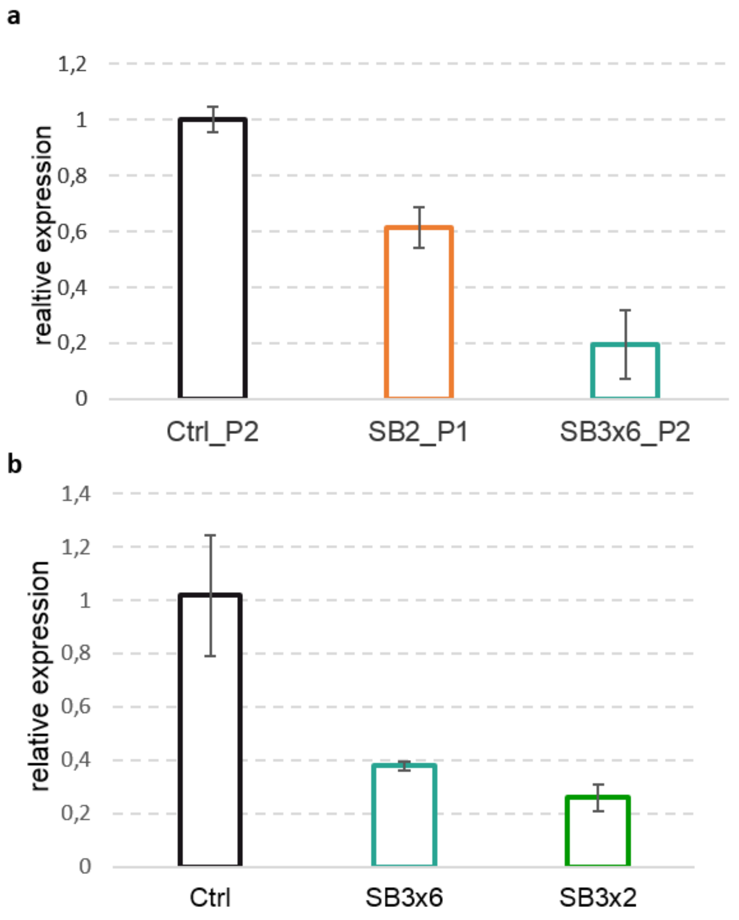

Supplementary Figure 3: Relative expression of *slaB* mRNA of silenced and control cultures measured by RT-qPCR. **a)** Relative *slaB* copies normalized to 16S rRNA reference gene of cultures transfected with pDEST-SB constructs, sampled at OD_{600}= 0.1 (subset of same cultures as in main paper). Error bars, ± *SD* three techn. repl). **b)** Relative amount of *slaB* mRNA copies (compared to SSOP1_3283 reference gene) of pIZ-SB-silenced cultures harvested at a higher OD_{600} of 0.2. Similar silencing levels are observed as in cultures sampled at OD_{600}= 0.1. Error bar, mean ± *SD* (three biol. replicates). Source data are provided[1].

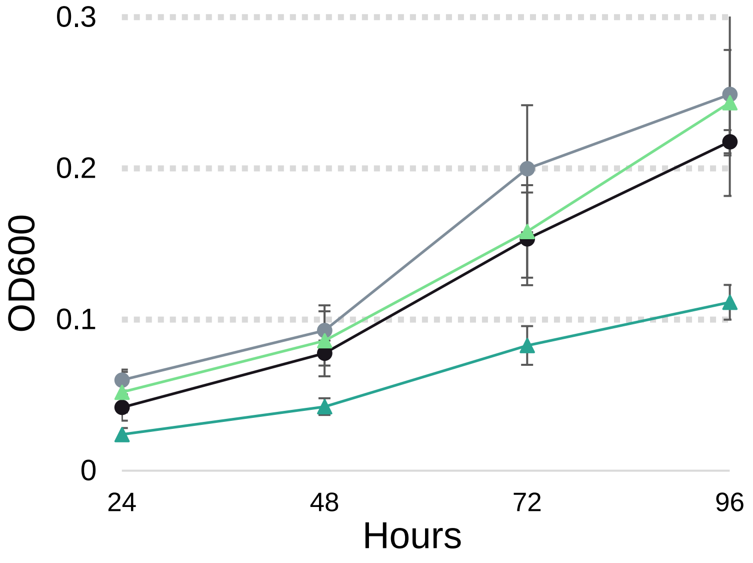

Supplementary Figure 4: Optical density increase of pDEST-SB plaque cultures carrying silencing construct SB3x6 (blue), control construct Ctrl (dark grey) and complementation constructs SB3x6-CC (light petrol), and Ctrl-CC (light grey), respectively. Error bars, mean ± *SD* (three biological replicates raised from three different plaques). Source data are provided[1].

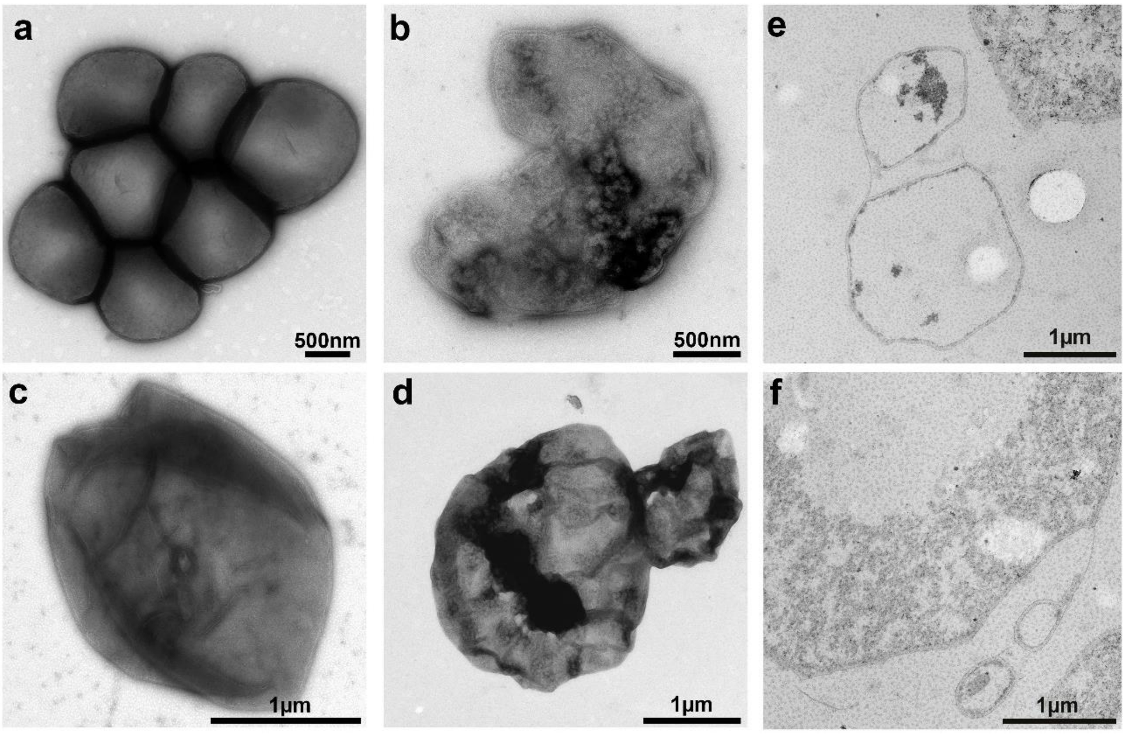

Supplementary Figure 5: Electron micrographs. Negatively stained (2% uranyl acetate) samples Ctrl **(a)** and 3x6 phenotypes **(b-d)**. **e-f)**: Thin sections of silenced SB3x6 cells (contrasted with Uranyl acetate 2%, and Lead-citrate 2%). Scale bars are given on the individual images.

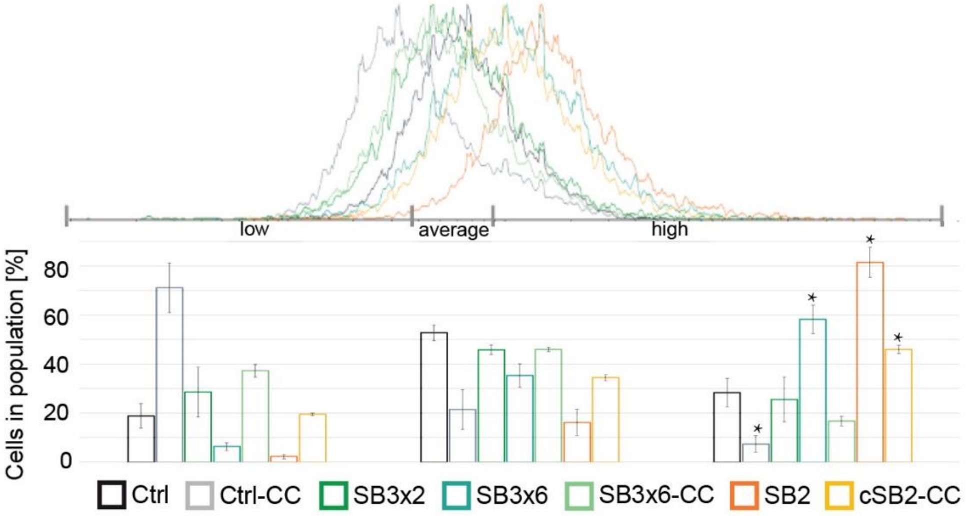

Supplementary Figure 6: SSC-histograms (side scatter, flow cytometry, created with *Flowing Software 2.5.1*) reflecting cell surface irregularity (3 biol. repl, *n* = 10000 per replicate). Bar chart represents relative abundance of cells in population showing low, average or high granularity relative to Ctrl. Error bars, mean ± *SD* (three biol. repl.). Asterisks indicate significant differences to Ctrl (two-tailed *t*-test, $n \geq 3$, $p \leq 0.02$). Source data are provided[1].

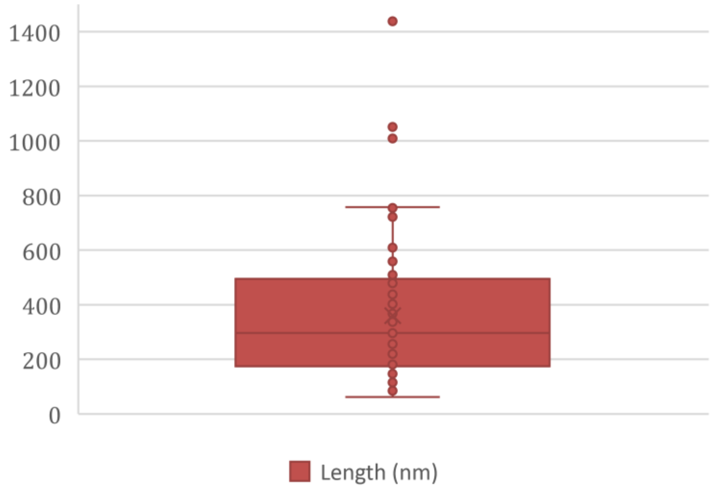

Supplementary Figure 7: Statistical analysis of the length of gaps in the S-layer, and detached S-layer fragments observed on electron micrographs of thin sectioned cells harvested from different transformation rounds at OD_{600}=0.1. Cell sizes were measured using ImageJ software (*n* = 55). Line=median, x= mean. Source data are provided[1].

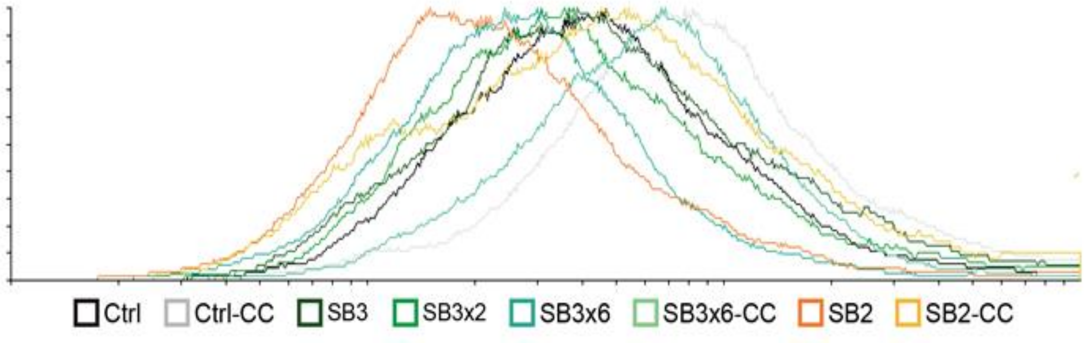

Supplementary Figure 8: Fluorescence-histograms (created with *Flowing Software 2.5.1*) reflecting the glycosylation differences in various cultures compared to the cells size (3 biol. repl, *n* = 10000 per replicate). The samples were stained with WGA-Alexa488 (Thermo Fisher Scientific) and analysed (FACSCanto II, BD). In order to normalize the measure fluorescence to cell size, the FITC-H measurements for each cell were divided by the FSC-A measurements for each cell, resulting in a FITC-H FSC-A ratio. For the sake of visualization, the obtained values were multiplied by a factor of 50 and plotted on a log scale. Source data are provided[1].

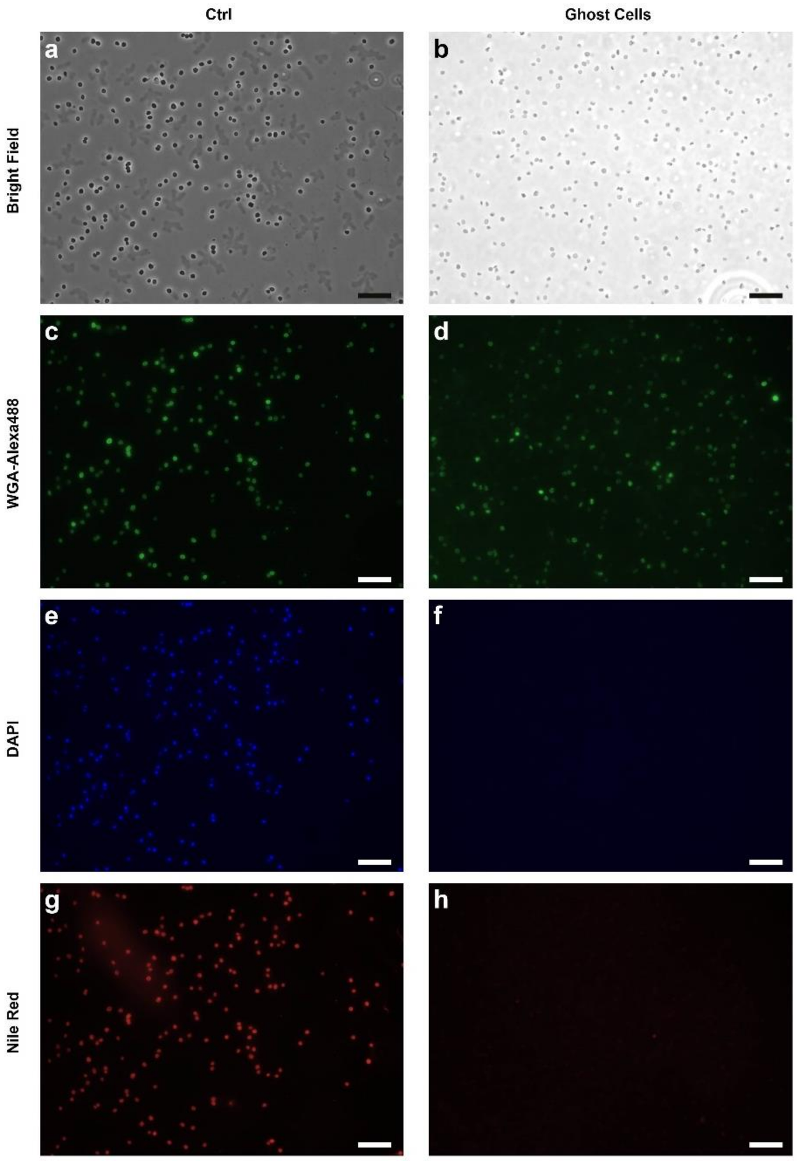

Supplementary Figure 9: Light microscopy and Fluorescent micrographs of freshly harvested Ctrl cultures and Ghost cells produced from freshly harvested Ctrl cultures (OD_{600}=0.1). bar= 10µm. **a-b)** represent phase contrast images of Ctrl **(a)** and Ghost **(b)** cells. **c-h)** show Fluorescent micrographs of cells stained with indicated dyes. Lectin stain (WGA, Alexa Fluor™ 488 Conjugate, Thermo Fisher Scientific) gave signal in ghost incubations which verified binding to S-layer.

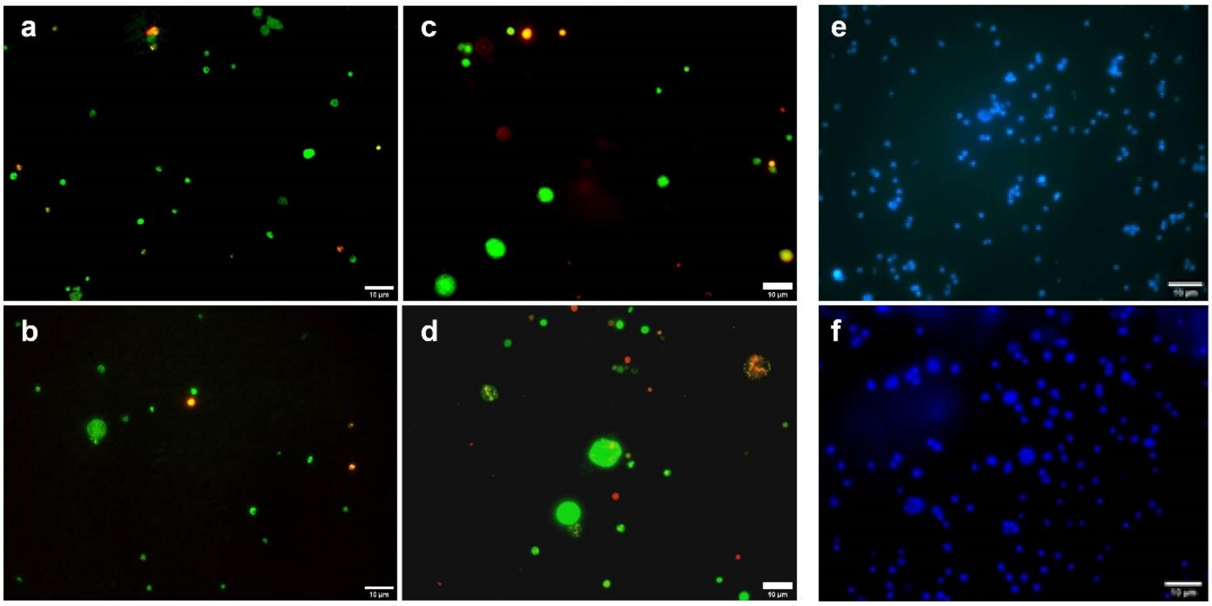

Supplementary Figure 10: Fluorescent micrographs of freshly harvested and stained pIZ-plasmid cultures from different transformation rounds at OD_{600}=0.1. **a, c)** LIVE/DEAD stain of SB3x6 cultures, **b, d)** LIVE/DEAD stain SB2 cultures. **e)** DNA-DAPI stain of a SB3x6 culture, **f)** DNA-DAPI stain of a SB2 culture. Scale bar=10µm.

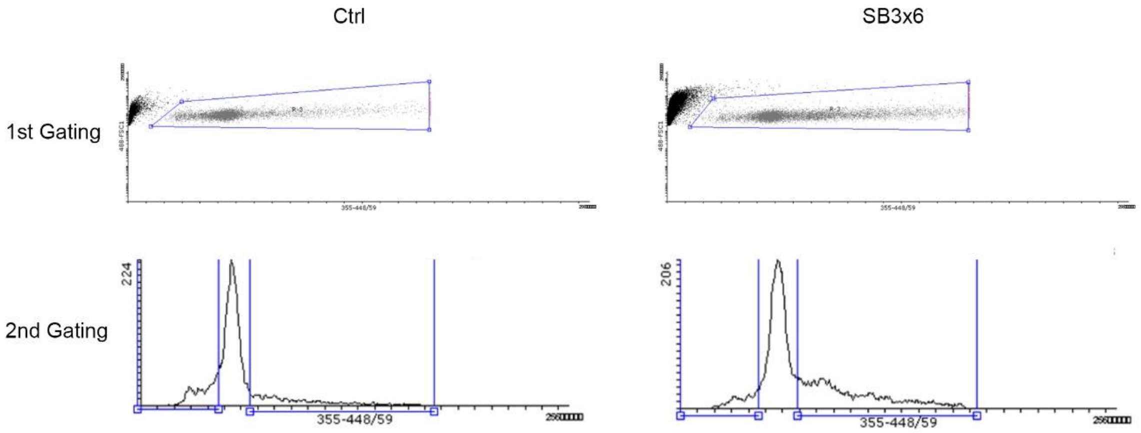

Supplementary Figure 11: Examples of FACS gating of Hoechst stained cells. Left: Ctrl; Right: SB3x6; Top: Dotplot showing which datapoints were used for further analysis (cf. Fig 6b in main manuscript); Bottom: Showing the three regions used for statistical comparison.

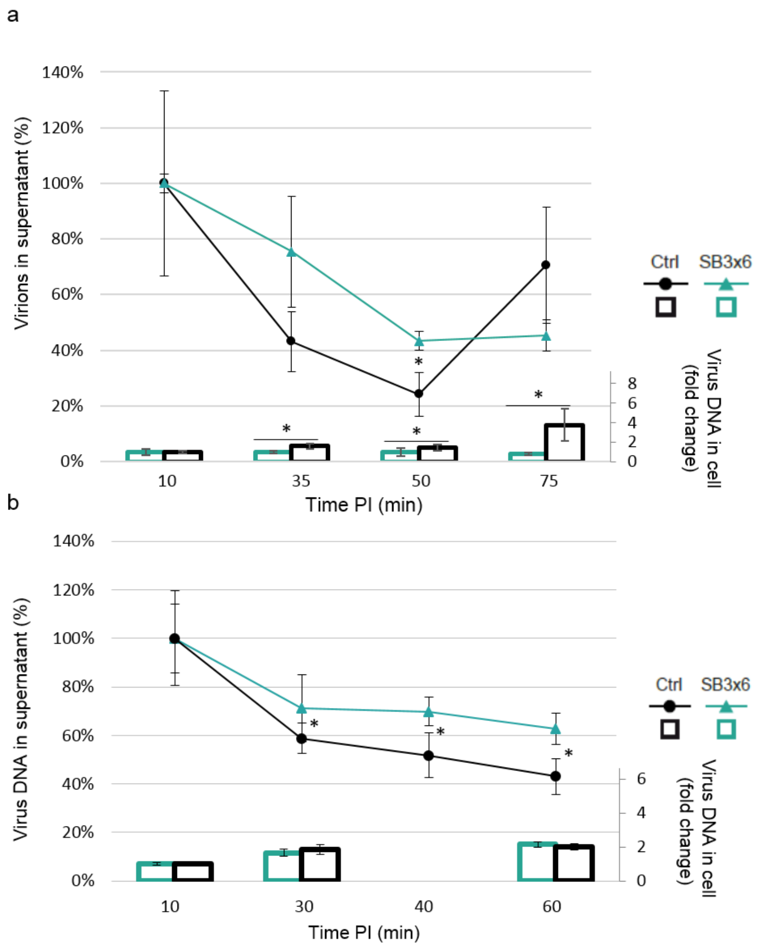

Supplementary Figure 12: Change of extracellular and intracellular SSV1 amount in SB3x6 and Ctrl cultures at different timepoints postinfection (PI) and different MOIs compared to 10 min PI. **a) MOI 5:** Extracellular virions determined by plaque assay (PFU per ml) compared to cell-free virus control (lines). Intracellular increase of virus-DNA in the same cultures measured by qPCR (bar charts). Significant differences between SB3x6 and Ctrl cultures were determined by two-tailed *t*-test, $n = 3$, $p \leq 0.00987$ (intracellular) and $p \leq 0.0369$ (extracellular) indicated with an asterisk. Error bars, mean ± *SD* (three biol. repl. and techn. Repl.). **b) MOI >100:** Extracellular (lines) and intracellular (bar chart) virus DNA increase and decrease, respectively, were determined by qPCR. Significant differences between SB3x6 and Ctrl cultures were determined by two-tailed *t*-test, $n \geq 3$, $p \leq 0.04623$ (extracellular) indicated with an asterisk. Error bars: as above. Source data are provided[1].

Supplementary Table 1: Primer and Spacer sequences used in this study.

qSlaBFW	TGCTACTGGTGGAATTTATGTGCCC
qSlaBFW	TGCTACTGGTGGAATTTATGTGCCC
qSlaBRV	TTGGAGCTACTGCGGGAGTTGT
q3194FW	ATCAGTGGAGACGAGTGGCAAGA
q3194RV	ATTGCAGCCTTAACCTCGCCTTCT
16SFW	TTGGGATCGAGGGCTGAAAC
16SRV	CTCACCCCTCTCCTACTCGG
M_Fw	TGCAGAATTATCGCCCAGAACAA
M_Rv	GTTAGTTCACCCACCGACAAATACA
CTRL-FW	AGAATTATCGCCCAGAACAAATTTCTGATAATCTCTTATAGAATTGAAAG
CTRL-RV	GTTAGTTCACCCACCGACAAATACAACTTTCAATTCTATAAGAGATTATC
miniCR-SB1FW	ATA AGT TTA TAA CAG CTC CTT GAC CAG CAG ATA ATC TCT TAT AGA ATT GA-
miniCR-SB1RV	AAGGAGCTGTTATAAACTTATTCTTCCCTCTTTCAATTCTATAAGAGATT
miniCR-SB2FW	CATTAGCAAACTCTATAAACTGTGGGTAAGATAATCTCTTATAGAATTGA
miniCR-SB2RV	AGTTTATAGAGTTTGCTAATGGTATCATACTTTCAATTCTATAAGAGATT
miniCR-SB3FW	AACGACCCGTTTGAAGCAGTTGTCGTAGAGATAATCTCTTATAGAATTGA
miniCR-SB3RV	AACTGCTTCAAACGGGTCGTTCATGTTAACTTTCAATTCTATAAGAGATT
SlaB3_Lin_FW	ACGGGTCGTTCATGTTAACTTT
SlaB3_Lin_RV	TTGAAGCAGTTGTCGTAGAGATAA
SlaB3_duplex_FW	TAAGAGATTATCTCTACGACAACTGCTTCAAACGGGTCGTTCATGTTAACTTT
SlaB3_duplex_RV	TAGAATTGAAAGTTAACATGAACGACCCGTTTGAAGCAGTTGTCGTAGAGATAA
SlaB3_triplex_FW	ACGGGTCGTTCATGTTAACTTTCAATTCTATAAGAGATTATCTCTACGACAACTGCTTC
SlaB3_triplex_ RV	ACGGGTCGTTCATGTTAACTTTCAATTCTATAAGAGATTATCTCTACGACAACTGCTTCAAACGGGTCGTTCATGTTAA
SlaBM164_FW	ATTTGAGGTGAATGGTTATGAAAAA
SlaBM164_SalI_RV	aagcgtcgacTTTATTTTTTTCTTCAAACCAATAGC
araFW_EagI	gaaacggccgGATCTTAGATAATCTGAGTTTGATCTTTTATGT
araOH_MSlaB_RV	TTTTTCATAACCATTCACCTCAAATcgggtacttttatgacctaactattttataa
406-FW	ATAAGTTTGATGGGGCAGCA
406-RV	TGACCATGATTACGAATTCGA
ORF-904FW	ACAAGAAGAACGGGGGTG
ORF 904RV	ACCTCTTCAGCAATCGCCT
qD291FW	ACTATAGCCTTAACGCAGAAGGGT
qD291RV	TAGTTGTGTGCCCGCAAAACTG

Supplementary References

1. Zink, I. A., Pfeifer, K. & Schleper, C. ZinkPfeifer_Source_Data.xlsx. (2019). doi:10.6084/m9.figshare.9772511.v1
