# Supplementary material for: CRISPR-mediated gene silencing reveals involvement of the archaeal S-layer in cell division and virus infection
Source: Nat Commun. 2019 Oct 22;10:4797. doi: 10.1038/s41467-019-12745-x (PMC6805947; doi:10.1038/s41467-019-12745-x)
Supplement: Supplementary file 3 — Reporting Summary [file 41467_2019_12745_MOESM3_ESM.pdf]

nature research

Corresponding author(s): Christa Schleper

Last updated by author(s): Sep 5, 2019

Reporting Summary

Nature Research wishes to improve the reproducibility of the work that we publish. This form provides structure for consistency and transparency in reporting. For further information on Nature Research policies, see Authors & Referees and the Editorial Policy Checklist.

Statistics

For all statistical analyses, confirm that the following items are present in the figure legend, table legend, main text, or Methods section.

n/a	Confirmed	
☐	☒	The exact sample size (*n*) for each experimental group/condition, given as a discrete number and unit of measurement
☐	☒	A statement on whether measurements were taken from distinct samples or whether the same sample was measured repeatedly
☐	☒	The statistical test(s) used AND whether they are one- or two-sided<br>*Only common tests should be described solely by name; describe more complex techniques in the Methods section.*
☒	☐	A description of all covariates tested
☒	☐	A description of any assumptions or corrections, such as tests of normality and adjustment for multiple comparisons
☐	☒	A full description of the statistical parameters including central tendency (e.g. means) or other basic estimates (e.g. regression coefficient) AND variation (e.g. standard deviation) or associated estimates of uncertainty (e.g. confidence intervals)
☒	☐	For null hypothesis testing, the test statistic (e.g. *F*, *t*, *r*) with confidence intervals, effect sizes, degrees of freedom and *P* value noted<br>*Give P values as exact values whenever suitable.*
☒	☐	For Bayesian analysis, information on the choice of priors and Markov chain Monte Carlo settings
☒	☐	For hierarchical and complex designs, identification of the appropriate level for tests and full reporting of outcomes
☒	☐	Estimates of effect sizes (e.g. Cohen's *d*, Pearson's *r*), indicating how they were calculated

Our web collection on statistics for biologists contains articles on many of the points above.

Software and code

Policy information about availability of computer code

Data collection

Provide a description of all commercial, open source and custom code used to collect the data in this study, specifying the version used OR state that no software was used.

Data analysis

Provide a description of all commercial, open source and custom code used to analyse the data in this study, specifying the version used OR state that no software was used.

For manuscripts utilizing custom algorithms or software that are central to the research but not yet described in published literature, software must be made available to editors/reviewers. We strongly encourage code deposition in a community repository (e.g. GitHub). See the Nature Research guidelines for submitting code & software for further information.

Data

Policy information about availability of data

All manuscripts must include a data availability statement. This statement should provide the following information, where applicable:

- Accession codes, unique identifiers, or web links for publicly available datasets
- A list of figures that have associated raw data
- A description of any restrictions on data availability

The source data underlying Figs 1b-f, 2c-e, 4, 5b-d, 6a, 6c, 7b Table1 as well as for Supplementary Figs Figures 3a-b, 4, 7, 11 and 12a-b are provided as a Source Data file, available in the “figshare” repository (doi:10.6084/m9.figshare.9772511.v1)

Field-specific reporting

Please select the one below that is the best fit for your research. If you are not sure, read the appropriate sections before making your selection.

☒ Life sciences ☐ Behavioural & social sciences ☐ Ecological, evolutionary & environmental sciences

For a reference copy of the document with all sections, see nature.com/documents/nr-reporting-summary-flat.pdf

Life sciences study design

All studies must disclose on these points even when the disclosure is negative.

Sample size	As three biological replicates are common practice in prokaryotic in vivo research, sample size was determined accordingly. We have repeated each experiment which has repeatedly shown the same trend.
Data exclusions	Data were only excluded when a technical problem in the measuring machine was detected. If possible, measurement of these data was repeated.
Replication	We have extensively replicated every experiment in this study and could reproduce the presented data.
Randomization	For cell size determination though light microscopy, positions on the slide were chosen at random. Plates and liquid cultures were incubated in random order to avoid influence of uneven heat distribution.
Blinding	Samples for Light and Electron Microscopy were given numbers instead of names to ensure unbiased observartions.

Reporting for specific materials, systems and methods

We require information from authors about some types of materials, experimental systems and methods used in many studies. Here, indicate whether each material, system or method listed is relevant to your study. If you are not sure if a list item applies to your research, read the appropriate section before selecting a response.

Materials & experimental systems

n/a	Involved in the study	
☒	☐	Antibodies
☒	☐	Eukaryotic cell lines
☒	☐	Palaeontology
☒	☐	Animals and other organisms
☒	☐	Human research participants
☒	☐	Clinical data

Methods

n/a	Involved in the study	
☒	☐	ChIP-seq
☐	☒	Flow cytometry
☒	☐	MRI-based neuroimaging

Flow Cytometry

Plots

Confirm that:

☒ The axis labels state the marker and fluorochrome used (e.g. CD4-FITC).

☒ The axis scales are clearly visible. Include numbers along axes only for bottom left plot of group (a 'group' is an analysis of identical markers).

☒ All plots are contour plots with outliers or pseudocolor plots.

☒ A numerical value for number of cells or percentage (with statistics) is provided.

Methodology

Sample preparation	Cell-Size (FSC), cell granularity (SSC) and glycoprotein (FITC) analysis was performed on a BD FACSCantoII. For FSC and SSC measurements, 50µl of cell from a growing culture were diluted 1:10 in Buffer A. For glycoprotein quantification, 20µl of freshly harvested cells were diluted 1:20 in Buffer A and subsequently incubated with 40µg/ml Wheat Germ Agglutinin, Alexa Fluor™ 488 Conjugate (W11261, Thermo Fisher Scientific) for 30 minutes in the dark at room temperature and analysed. To measure the DNA content, cells were fixed at 4°C over night in 70% ice cold ethanol, subsequently washed and resuspended in Buffer A and stained with Hoechst 33342 (Invitrogen H1399) according to the manufacturers protocol. 50µl of the stained cells were diluted 1:10 in the washing buffer and analyzed (MoFlo Astrios. Beckman Coulter).
Instrument	MoFlo Astrios. Beckman Coulter, BD FACSCantoII

Software	Flowing Software 2.5.1 (Perttu Terho)
Cell population abundance	We did not perform cell sorting.
Gating strategy	First Gating was performed by setting FSC and SSC cut-off values (800 and 200 respectively). Cells stained with Syto9 (ThermoFisher Scientific) were measured to optimize these cut-off values and to discriminate against debris. Gating for DNA content determination is shown in Supplementary Material.

☒ Tick this box to confirm that a figure exemplifying the gating strategy is provided in the Supplementary Information.
